# Kinect-Based Correction of Overexposure Artifacts in Knee Imaging with C-Arm CT Systems

**DOI:** 10.1155/2016/2502486

**Published:** 2016-07-19

**Authors:** Johannes Rausch, Andreas Maier, Rebecca Fahrig, Jang-Hwan Choi, Waldo Hinshaw, Frank Schebesch, Sven Haase, Jakob Wasza, Joachim Hornegger, Christian Riess

**Affiliations:** ^1^Pattern Recognition Laboratory, Computer Science Department 5, University of Erlangen-Nuremberg, 91058 Erlangen, Germany; ^2^Department of Informatics, Technical University of Munich, 85748 Garching bei München, Germany; ^3^Department of Radiology, Stanford University, Stanford, CA 94305, USA

## Abstract

*Objective.* To demonstrate a novel approach of compensating overexposure artifacts in CT scans of the knees without attaching any supporting appliances to the patient. C-Arm CT systems offer the opportunity to perform weight-bearing knee scans on standing patients to diagnose diseases like osteoarthritis. However, one serious issue is overexposure of the detector in regions close to the patella, which can not be tackled with common techniques.* Methods.* A Kinect camera is used to algorithmically remove overexposure artifacts close to the knee surface. Overexposed near-surface knee regions are corrected by extrapolating the absorption values from more reliable projection data. To achieve this, we develop a cross-calibration procedure to transform surface points from the Kinect to CT voxel coordinates.* Results.* Artifacts at both knee phantoms are reduced significantly in the reconstructed data and a major part of the truncated regions is restored.* Conclusion.* The results emphasize the feasibility of the proposed approach. The accuracy of the cross-calibration procedure can be increased to further improve correction results.* Significance.* The correction method can be extended to a multi-Kinect setup for use in real-world scenarios. Using depth cameras does not require prior scans and offers the possibility of a temporally synchronized correction of overexposure artifacts. To achieve this, we develop a cross-calibration procedure to transform surface points from the Kinect to CT voxel coordinates.

## 1. Introduction

**Figure 1:**
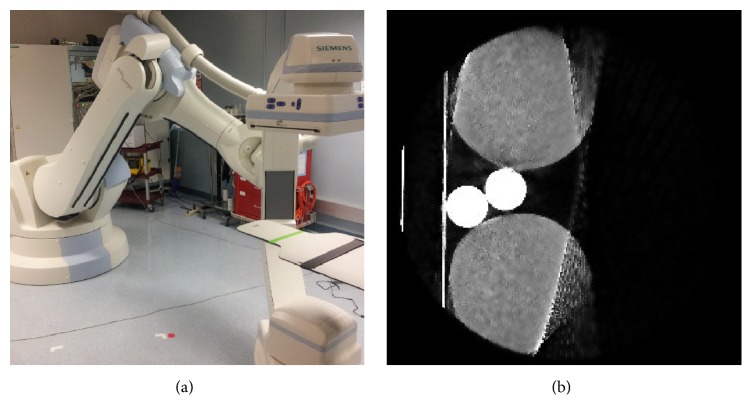
(a) The Siemens Zeego C-arm CT system. The robotic arm allows free movement of the C-arm for scanning patients in standing position. (b) Typical artifacts that arise when scanning knee-shaped objects with a C-arm CT. Due to saturation, the original cylindrical shape is lost, and the front is severely affected by artifacts (window level [−1000, 1500]).

C-arm CT systems (Figure 1(a)), in contrast to conventional CT systems, have a high mechanical flexibility which gives radiologists the opportunity to perform CT scans in a variety of spatial positions. In particular, it is possible to rotate the CT system around a vertical axis [1]. This enables imaging of patients with knee diseases such as osteoarthritis while they are standing in an upright position, hence while the knee is bearing the weight of the patient [2].

One challenge of imaging relatively thin body parts like the knee is the limited dynamic range of the C-arm CT flat panel detector, leading to overexposure of the exterior regions of the knee. If not avoided or compensated for, overexposure leads to artifacts in the reconstructed volume, as shown in Figure 1(b). The front and back of the knee appear blurry and lack clearly defined outer boundaries. The image quality of important parts of the knee image, such as the patella, is severely affected by these artifacts. This has a negative impact on reliability of the diagnosis.

Using a C-arm CT acquisition protocol with the patient lying in supine position, several approaches are available to avoid or compensate overexposure artifacts. One way to avoid overexposure artifacts during acquisition is by covering the knees with an additional absorber, for example, a rubber belt [2, 3]. However, extra weight of the belt can cause great discomfort for an upright patient with pains in the knees.

Different algorithmic methods for truncation correction in C-arm CT systems have been developed in the recent years. Truncation artifacts that arise in scans with a small region of interest can be effectively corrected without any explicit extrapolation scheme [4]. If bigger portions of the patient are of diagnostic interest, different correction methods have to be applied. In [5], additional knowledge through a prior low-intensity scan is facilitated for artifact correction. In the case of imaging of standing patients with knee diseases, however, expected patient movement makes the use of a prior scan very difficult.

Other methods, which do not use a prior low-intensity scan, correct truncation artifacts through an appropriate extrapolation model such as a water cylinder for the upper body [6, 7]. In [8], the model-based extrapolation is extended by an iterative truncation correction algorithm, which is able to handle cases where the water cylinder assumptions are not exactly fulfilled. These model-based methods are not applicable for knee imaging, as the anatomical structure is too complex to be approximated by a single cylindrical or elliptical object. Another approach which uses a multicylinder extrapolation model [9] yields better results. Similar to the single water cylinder model, however, overexposure correction only works for objects that sufficiently fit to the simplified cylindrical knee models.

Hence, in order to bring the novel diagnostic possibility of imaging knees of standing patients into clinical practice, it is highly desirable to develop an imaging solution that avoids these drawbacks.

In this paper, we present a method for correcting overexposure by combining information from a Kinect depth camera with a C-arm system. As a proof of concept, we demonstrate its feasibility for patients in supine position. However, there is no fundamental limitation for applying the same setup to patients in weight-bearing standing position. In such scenarios, multiple Kinect depth cameras, observing the patient from different angles, could be used for artifact correction. The approach has the further advantage that the information used for correction can be acquired simultaneously to the CT scan. Thus, depreciation of the correction through patient movement is low in comparison to methods relying on prior information.

The contributions of the paper are as follows:

- We introduce a specifically designed, easy-to-reproduce calibration target for cross-calibrating a C-arm CT system with a Kinect depth camera.
- We propose a cross-calibration procedure between the depth camera and the C-arm CT.
- We present a depth-based correction of overexposure artifacts.

**Figure 2:**
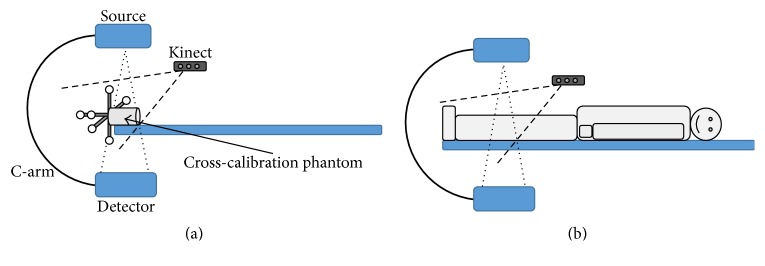
(a) A Kinect camera is cross-calibrated to the C-arm CT using a phantom on the patient bench. (b) For overexposure correction, the patient is imaged simultaneously by the C-arm and the Kinect.

Figure 2(a) shows a sketch of the cross-calibration procedure using a calibration phantom. The calibration target is detected by both imaging systems and enables the computation of a transformation of the coordinates from one modality to coordinates of the other modality.

Figure 2(b) shows a sketch of the imaging protocol. Once the system is calibrated, a patient is placed into the field of view of both modalities.

When imaging a patient, the Kinect depth data is used to find the points of intersection between the X-ray beam path and the object surface, that is, the points at which the X-rays enter and leave the knee tissue. For each pixel in each projection, the length of the beam path across the knee is calculated. Overexposed pixels are corrected by extrapolating the absorption along the corresponding line integrals.

In Section 2, we describe the phantom and the cross-calibration procedure for transforming points between both imaging modalities. In Section 3, we describe the proposed projection-based artifact correction. In Section 4, the reconstruction of the corrected projections is evaluated and compared with an uncorrected volume and the ground truth. In Section 5, we discuss the correction results and limitations of our proposed method. In Section 6, we discuss possible improvements and future work based on the current correction method.

## 2. Kinect to CT System Cross-Calibration

The Microsoft Kinect camera provides a color image and additionally per pixel the distance in 3D of the depicted scene point to the camera. To use this distance information in a CT scan, we determine the parameters for a rigid transformation between both imaging systems through cross-calibration procedure.

A cross-calibration phantom with known geometry is observed by both imaging modalities to determine the relative translation and rotation between both coordinate systems.

**Figure 3:**
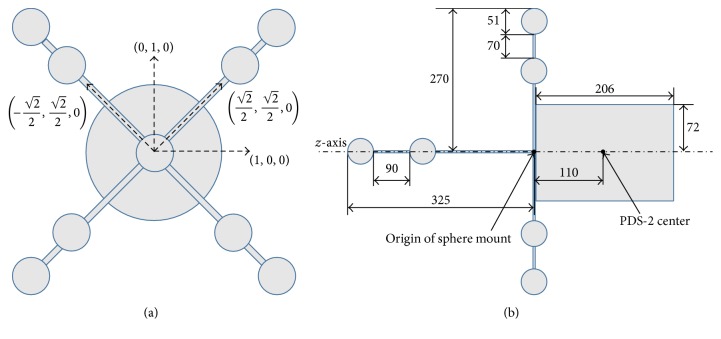
(a) Relative alignment of the predefined axes of the cylindrical PDS-2 phantom and the depth calibration structure with the unit direction vectors in the Zeego coordinate system. (b) The origin of the depth calibration structure aligns with the center of the PDS-2 phantom along the *z*-axis.

The cross-calibration phantom consists of the cylindrical PDS-2 calibration phantom, which is commonly used for C-arm cone-beam CT calibration [10], and an attached depth calibration structure.  Figure 3 shows the basic design and geometry of the phantom. The depth calibration structure is a scaffold of orthogonal plastic rods.

Three spheres are attached on each rod. The spheres are particularly suitable for detection and localization with the Kinect camera from a wide range of viewing angles. The goal of the calibration is to identify the three rods with the coordinate axes and their intersection with the coordinate origin.

From solely observing the cylinder surface, only the direction of the axis of the cylinder could be determined. The spheres allow the determination of the alignment of the *x*- and *y*-axes purely based on depth data. Painting the cylinder to indicate the axes directions, for example, would introduce inaccuracies from the Kinect-internal RGB-to-depth calibration to the cross-calibration procedure.

The use of the attachment could prove to be especially advantageous in weight-bearing scanning scenarios, where two or more Kinect cameras are observing the phantom from different angles for the calibration.

**Figure 4:**
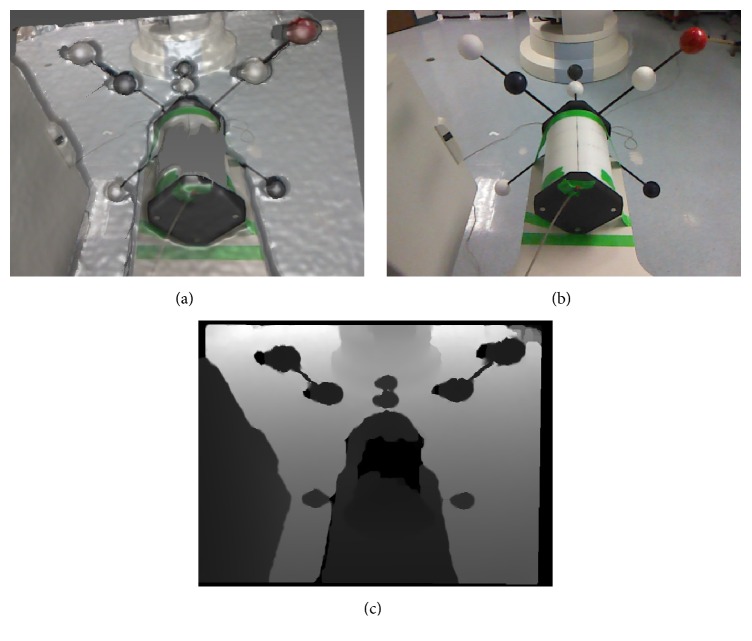
(a) shows an overlay of the depth and RGB data as captured by the Kinect camera. (b) and (c) show the separate RGB and depth images as used for this work.

For processing the depth data, we use the Range Imaging Toolkit RITK [11]. A visualization of acquired data can be seen in Figure 4. Raw depth images from the Kinect camera are relatively noisy, with a standard deviation of point-to-plane distances of about 25 mm at 1 m distance [12]. To counter this noise, we apply spatial smoothing (Gaussian kernel with *σ* = 2.5), temporal averaging (20 frames) and edge-preserving smoothing (guided filter with 4-pixel support [13]).

### 2.1. Sphere Segmentation and Fitting

First, a user has to mark the spheres in the RGB data. We compute the estimated projected size of the sphere from the depth information at the marked point. Pixels of similar depth around the seed point are recursively added to the sphere area, as long as the distance of newly added pixels does not exceed the sphere size.

**Figure 5:**
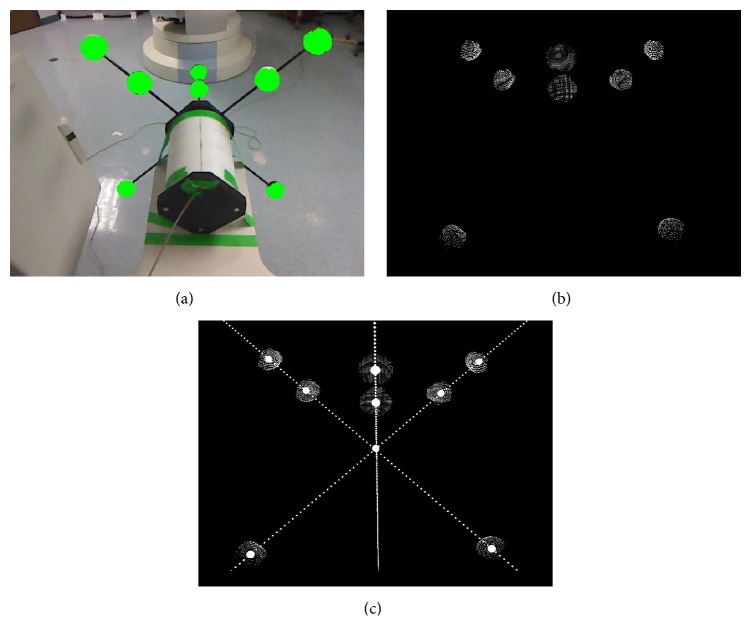
(a) The green areas visualize the resulting segmentation in the RGB image. (b) Surface points of the segmented pixels. (c) Sphere centers and subsequently the coordinate axes and origin are fitted to these points.

Spheres belonging to the same axis are fitted to the depth data. We estimate the sphere center for each connected set of sphere surface points (see Figure 5). An initial estimation of the sphere center is made by using *x* and *y* coordinates from the initially user-selected spheres. The depth value of the center is approximated by adding half of the sphere radius to the mean depth value of the respective surface points. The best fitting center point is determined using a least squares error metric. Let **p**_*i*_, 1 ≤ *i* ≤ *n*, be the *i*th surface point in Kinect 3D coordinates and **c** ^*∗*^ the unknown Kinect 3D center coordinate of the sphere. Then, **c** ^*∗*^ is determined by solving the convex optimization problem

(1) $$\begin{array}{c} c^{*}=\underset{c}{\mathrm{argmin}}\overset{n_{p}}{\underset{i=1}{\sum }}{\left\|\;p_{i}-c\;\right\|}_{2}^{2}, \end{array}$$

where *n*_*p*_ denotes the number of segmented sphere surface pixels.

### 2.2. Estimation of the Axes Directions

From the estimated center points, position and direction of the axes are obtained as follows. Per axis we use at least two center points (each axis has 2 to 4 spheres, resp.). Without loss of generality, we aim to recover one point on the* first* coordinate axis and its direction, denoted as ${\bar{c}}_{1}$ and **v**_1_, respectively. Let *n*_*s*_ ∈ {2,3, 4} denote the number of segmented spheres on this axis and **c**_*j*_^*∗*^, 1 ≤ *j* ≤ *n*_*s*_, the center of the *j*th segmented sphere. The axis point ${\bar{c}}_{1}$ is the 3D mean coordinate of all **c**_*j*_^*∗*^:

(2) $$\begin{array}{c} {\bar{c}}_{1}=\frac{1}{n_{s}}\overset{n_{s}}{\underset{i=1}{\sum }}c_{j}^{*}. \end{array}$$

The algorithm for finding **v**_1_ is analogous to finding the best fitting plane to the points. We solve this problem via orthogonal distance regression and singular value decomposition (SVD) [14].

Let

(3) $$\begin{array}{c} A=\left(\begin{array}{c} {\left(c_{1}^{*}-{\bar{c}}_{1}\right)}^{T}\\ \vdots \\ {\left(c_{n_{s}}^{*}-{\bar{c}}_{1}\right)}^{T} \end{array}\right) \end{array}$$

be a zero-mean matrix containing the displacement vectors of the sphere centers **c**_*j*_^*∗*^ to the mean center coordinate ${\bar{c}}_{1}$. SVD yields a matrix factorization **A** = **U** **S** **V** ^T^, where **S** is a diagonal matrix containing the singular values of **A**, and the columns of **U** and **V** are, respectively, left- and right-singular vectors corresponding to the singular values. Let **v**_1_ be the eigenvector in **V** associated with the largest singular value in **S**. Then,

(4) $$\begin{array}{c} a_{1}={\bar{c}}_{1}+t\cdot v_{1} \end{array}$$

is a least-square estimate of the first coordinate axis in parametric form with scale parameter *t*. Accordingly, we estimate other coordinate axes **a**_2_ and **a**_3_ from the two remaining sets of sphere centers.

### 2.3. Estimation of the Kinect Coordinate Origin

We calculate the axis origin as the estimated point of intersection of the rod axes. Due to noise and estimation inaccuracies, the axes are unlikely to intersect in one single point. Therefore, we define the coordinate origin as the closest point to all three axes in a least-squares sense [15].

The formula for calculating the closest point **g** to multiple *n*-dimensional lines is the following (see Appendix  A.1):

(5) $$\begin{array}{c} g={\left(\;\sum_{i=1}^{n}I-v_{i}v_{i}^{T}\;\right)}^{-1}\cdot \left(\;\overset{n}{\underset{i=1}{\sum }}\left(\;I-v_{i}v_{i}^{T}\;\right){\bar{c}}_{i}\;\right). \end{array}$$

The unit direction vectors **v**_*i*_ and suspension points ${\bar{c}}_{i}$ of the axes are already known from the previous estimation of the axes directions.

The solution **g** = (*x*_*g*_, *y*_*g*_, *z*_*g*_)^T^ is the fitted origin of the sphere mount in the Kinect coordinate system. All detected 3D points in the Kinect coordinate system are translated to the estimated origin **g**:

(6) $$\begin{array}{c} p^{{\text{Kinect}}^{\prime }}=\left[\begin{array}{c} x^{3D}\\ y^{3D}\\ z^{3D} \end{array}\right]-\left[\begin{array}{c} x_{g}\\ y_{g}\\ z_{g} \end{array}\right]. \end{array}$$

### 2.4. Coordinate System Transformation

Knowing the position and rotation of the calibration structure to the phantom, coordinates can be directly transformed from Kinect to the C-arm CT (see also Appendix  A.2). The coordinate system origin of the C-arm CT lies in the center of the cylinder (cf.  Figure 3(a)).

Let **W** capture rotation around *z*-axis and **t** ^Origins^ translation between *g* and the center of the cylinder. Then

(7) $$\begin{array}{c} p^{\text{Zeego}}=Wp^{{\text{Kinect}}^{\prime }}+t^{\text{Origins}} \end{array}$$

transforms a Kinect surface point **p** ^Kinect′^ into a C-arm CT coordinate **p** ^Zeego^.

## 3. Overexposure Artifact Correction

The flat panel detector used in C-arm CT imaging has a limited dynamic range. If both knees overlap in a projection, higher X-ray doses are necessary to penetrate both knees. In the exterior regions of the knees the X-rays are only slightly attenuated and the resulting high intensities at the detector cause saturation. Hence, information about these regions is lost and saturation artifacts arise.

### 3.1. Projection-Based Extrapolation

The correction of the saturation artifacts is performed for every detector line in each projection separately. Joint use of Kinect and CT data allows a straightforward correction of overexposure in three steps:

1. If a detector line in a projection contains overexposed pixels, we determine the 3D points where the X-rays entered and exited the knee.
2. From these points, the length of the beam path through the knee is computed.
3. Overexposed pixels are corrected by extrapolating a smooth absorption fall-off from nonoverexposed pixels.

Note that the extrapolation does not automatically suppress tissue variations at knee boundaries: the angular range in C-arm CT scans usually amounts to 200°. Upon tomographic reconstruction of the knee volume, there exist for each boundary voxel many projection angles where a sufficiently thick portion of the knee is traversed, such that tissue variations at knee boundaries can in principle still be observed.

### 3.2. Geometric Considerations of Correction

Figure 7(a) shows *x*-*z*-axis view of an X-ray beam hitting an exemplary detector line. We are interested in the length of the beam path through the knees.  Figure 7(b) shows the same trajectory in *x*-*y*-axis view. We are looking at rays on a plane defined by the X-ray source and the currently considered detector line. For each ray, we are seeking the intersection length of the ray with the knees.

**Figure 6:**
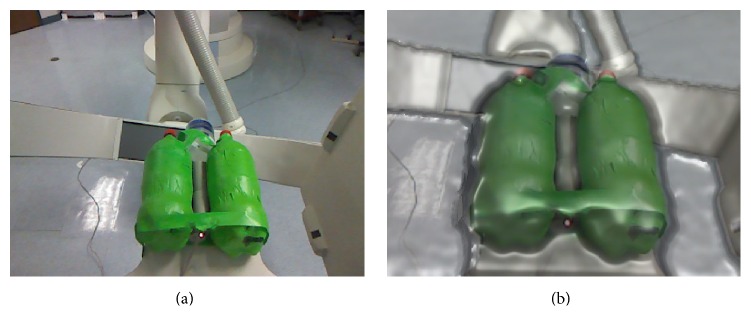
(a) shows an RGB image of the knee phantoms acquired by the Kinect camera. The corresponding preprocessed RGBD image can be seen in (b).

**Figure 7:**
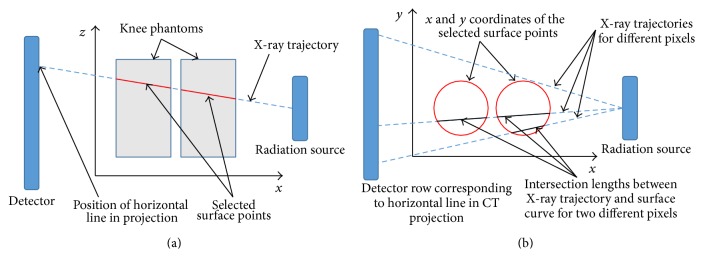
(a) The necessary surface points for the correction are selected by first disregarding the *y*-component of the points and the intersecting X-ray. Only surface points that have the same *z* coordinate as the trajectory line at their *x* coordinate are selected. (b) The calculation of the curve-line intersections is performed in 2D by only regarding the *x*- and *y*-values.

In our experiments, we simulate the knees with two plastic bottles filled with water (see Figure 6). To simulate the femurs in the legs in the CT images, two dense rods with a density of 1000 g/cm^2^ are placed between the bottles.

In principle, the intersection length can be directly computed from the nearest Kinect surface points at the entrance and exit of the knee. However, to make the results more robust to noise, we first fit a cubic B-spline curve to all points lying on the plane and determine the intersection length from the spline. Note that this computation can be performed in 2D, as all involved points are located on the same plane.

**Figure 8:**
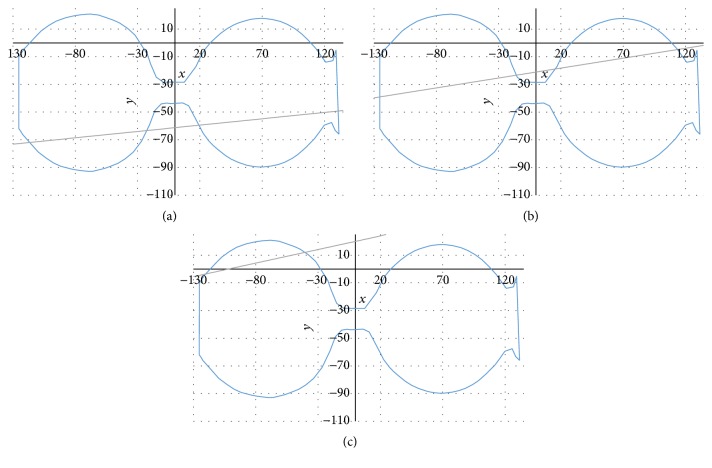
Examples for the B-spline interpolation of the surface points of two plastic bottles and different intersecting lines. The computed intersection lengths are (a) 209.91, (b) 213.25, and (c) 82.11.

Examples for resulting closed cubic B-spline curves are shown in Figure 8. Here, we observe two plastic bottles that represented the knees. The line that passes through the curve represents an example of the X-ray trajectory. In this case the *x*-component of the X-ray direction vector is dominating; that is, detector and radiation source lie close to the *x*-*z*-plane. Note the slight inaccuracies on the right side and truncated horizontal contours due to limitations in the edge detection of the depth camera. We extrapolated the surface points on the unobserved side of the knee phantoms by mirroring the visible points on a plane parallel to the *x*-*z*-plane.

**Figure 9:**
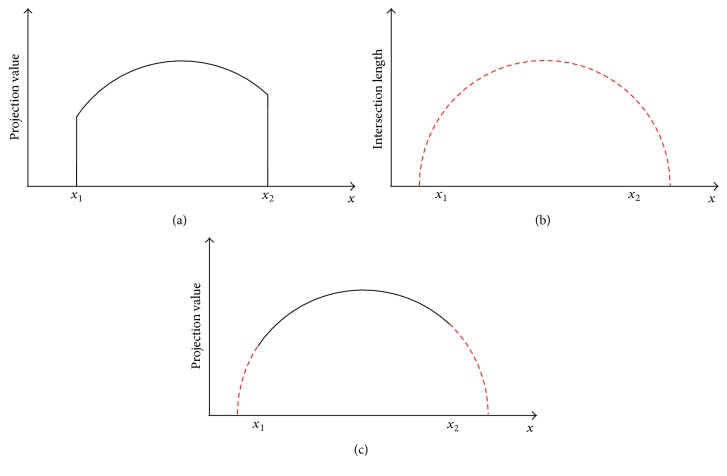
(a) shows exemplary values for an overexposed projection. Due to the saturation, there is no reliable information beyond the transition points to the overexposed regions *x*_1_ and *x*_2_. For correction, the calculated intersection lengths (b) are normalized to the projection value at *x*_1_ and *x*_2_ and used to extrapolate the boundary of the saturated object (c).

A schematic explanation of the proposed extrapolation method is shown in Figure 9. The objective is a smooth and reasonable extrapolation of the line integrals at the transitions to the overexposed regions *x*_1_ and *x*_2_.

For a smooth transition, the intersection lengths are normalized to match the value of line integral at *x*_1_ and *x*_2_, respectively. To prevent noise-related inaccuracies, we use an average value of the last nonoverexposed points for normalization.

**Figure 10:**
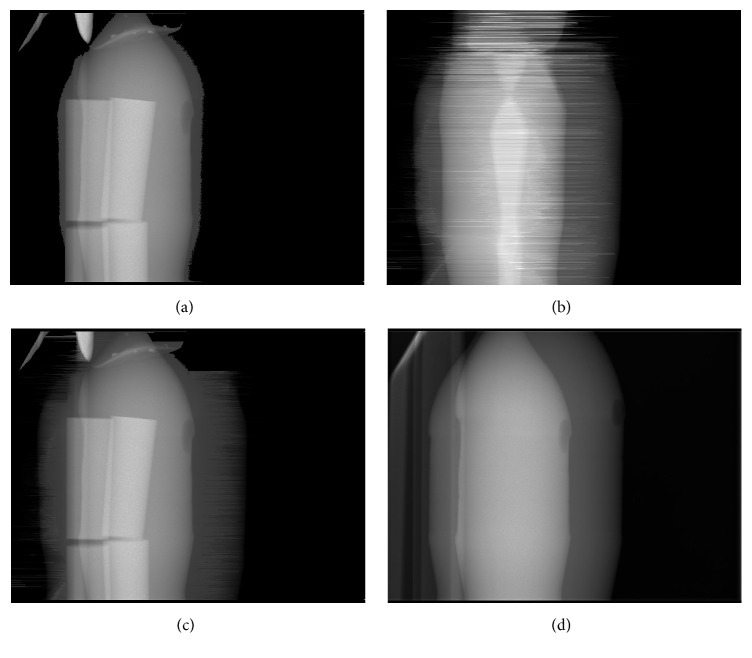
An uncorrected projection is shown in (a). (b) shows the intersection lengths that have been calculated for the projection geometry as in (a). Based on these intersection lengths, the saturated projection is extrapolated. The resulting corrected projection is illustrated in (c) and can be compared to the ground truth in (d). Window levels (a), (c), and (d): [0, 8]. Window level (b): [0, 300].

The result of the Kinect-based correction of a CT projection is demonstrated in Figure 10.

### 3.3. Reconstruction Setup

We use the CONRAD framework for reconstruction [16] after the artifact correction.

The reconstruction pipeline consists of a cosine weighting filter [17], a Parker redundancy weighting filter [18], a Shepp-Logan ramp filter [17], and a GPU-based back projection tool [19]. After the reconstruction, the data is normalized to the Hounsfield scale. In a final step, the reconstructed data is smoothed with a bilateral filter (width: 5, photometric distance: 500). The source-detector and source to *z*-axis distances are 1200 mm and 600 mm, respectively. We acquire 133 projections in a 200° rotation around the object. The detector size in pixels is 1240 × 960 with a pixel spacing of 0.308 mm for *x* and *y*. The mean distance of the Kinect camera to the phantom is 700 mm.

## 4. Results

We evaluate and compare the reconstructions of the four projection data sets which are shown in Figure 10. After a brief description of the reconstruction setup we describe the results for one slice of the reconstructed volumes.

Afterwards, the results are compared quantitatively for five regions of interest in the exterior region of the knee phantoms.

### 4.1. Observations

**Figure 11:**
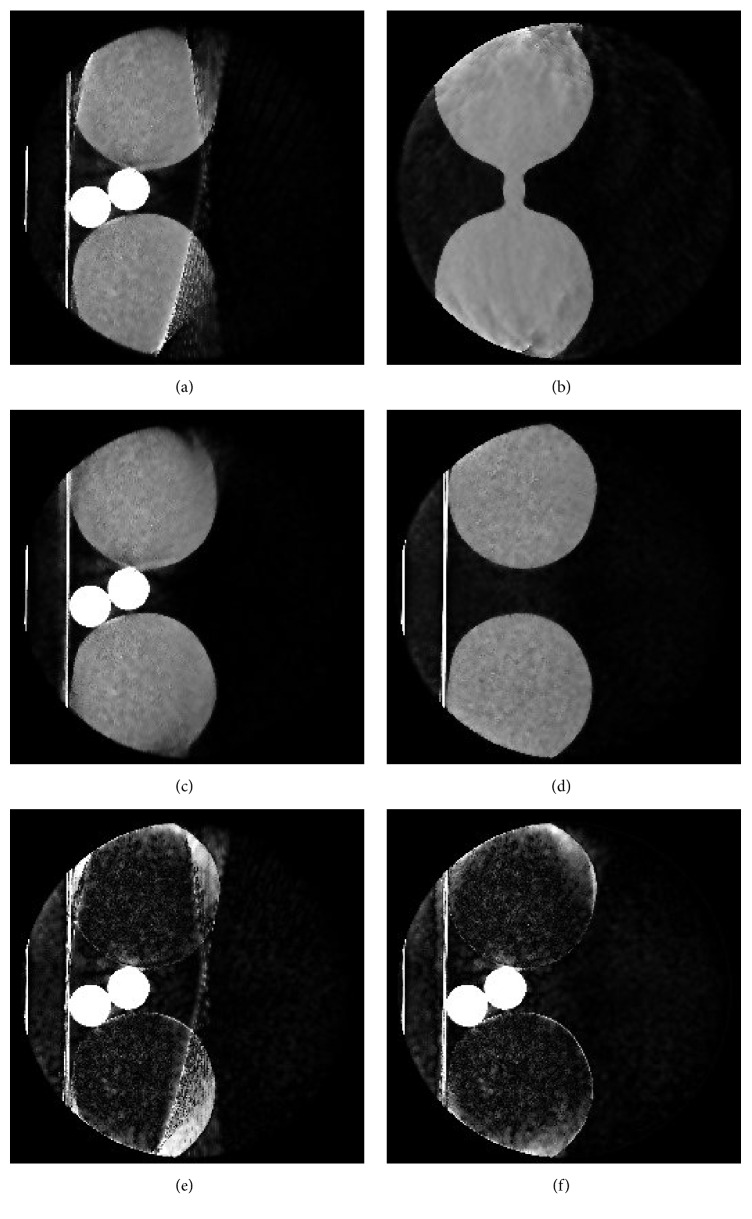
(a) shows a slice of the uncorrected volume. Severe artifacts deriving from saturation are visible on the right exterior regions of both bottles. (b) shows the corresponding slice to (a) which has been reconstructed by solely using the intersection lengths obtained from the depth data. These values are used for the correction of the saturation artifacts. A slice of the resulting corrected volume can be seen in (c). (d) shows the reconstruction of the nonoverexposed knee phantoms. The bone phantoms have been removed to acquire this ground truth reference. For further demonstration of the results, the absolute difference between the ground truth and the uncorrected data (e) and the corrected data (f) is visualized. The two dense rods between the two water cylinders have a density of 1000 g/cm^2^, simulating the femurs in the legs. Window level (a)–(d): [−1000, 1000]. Window level (e) and (f): [0, 1000].

We first inspect the reconstruction of the uncorrected projections (see Figure 11(a)). The saturation causes strong artifacts. High intensity streaks are observed at the onset of the overexposure and the original shape of the edges on the right side can not be clearly recognized. The exterior regions on the right side lack a definite outer boundary and are blurred.

Figure 11(c) shows the reconstruction of the corrected projections. The overexposure artifacts are significantly reduced for both bottles and the boundaries on the right side of the phantoms are mostly restored. However, the contour of the phantom is still blurred at the outer regions of the bottles in the top right and bottom right.

**Figure 12:**
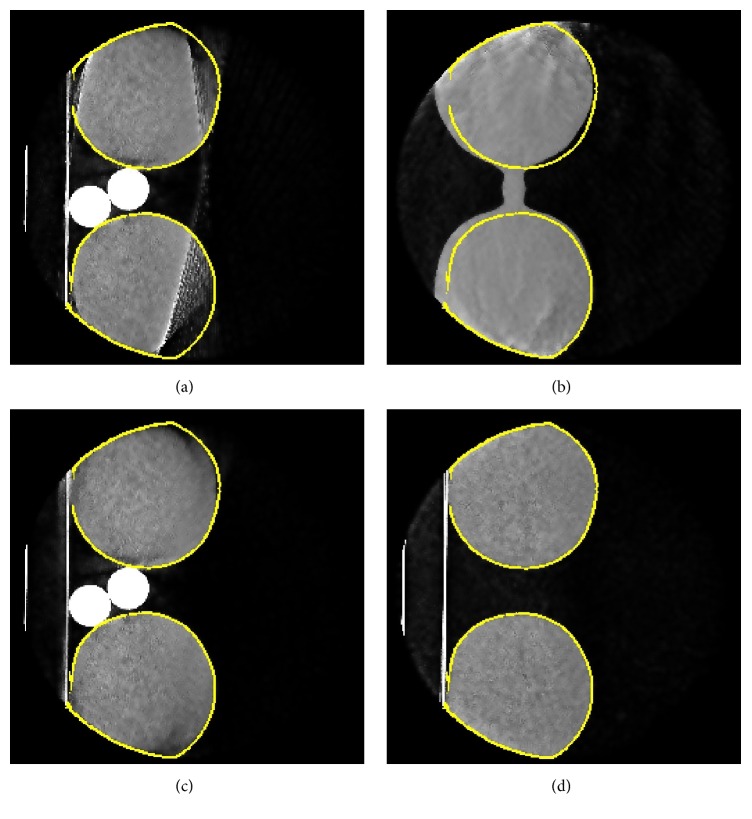
(d) shows the boundary of a slice created from the ground truth data. For comparison, this boundary is also shown in the corresponding slices of the uncorrected (a), corrected (c), and surface data (b). It can be observed that the edges on the right side of the surface data are not perfectly aligned with the ground truth. This results from inaccuracies in the cross-calibration. Window level: [−1000, 1000].

The boundaries of the ground truth and the surface data do not align perfectly (see Figure 12(b)). This problem arises from inaccuracies in the cross-calibration procedure. As these inaccuracies are sufficiently small, we can still achieve good correction results. The outline of the bottles in the left half of the surface data slice lies outside the ground truth boundary. This inaccuracy results from the extrapolation of surface points to the back side of the knees, which was based on mirroring the surface points on the *x*-*z*-plane at an estimated *y*-height.

In Figures 11 and 12 we observe that in principle there is sufficient depth information to extrapolate the truncated boundary within the field of view. However, the boundary was not restored completely in the corrected volume. The reason for this is the nonlinear preprocessing by the C-arm CT system. As a result of this preprocessing, the values of the last nonoverexposed pixels can be very low. If the intersection lengths are normalized to these very low values, the extrapolation is of almost no effect. This effect can be countered by starting the extrapolation at an earlier point at which the pixel values have not been minimized by preprocessing.

### 4.2. Quantitative Comparison of the Results

**Figure 13:**
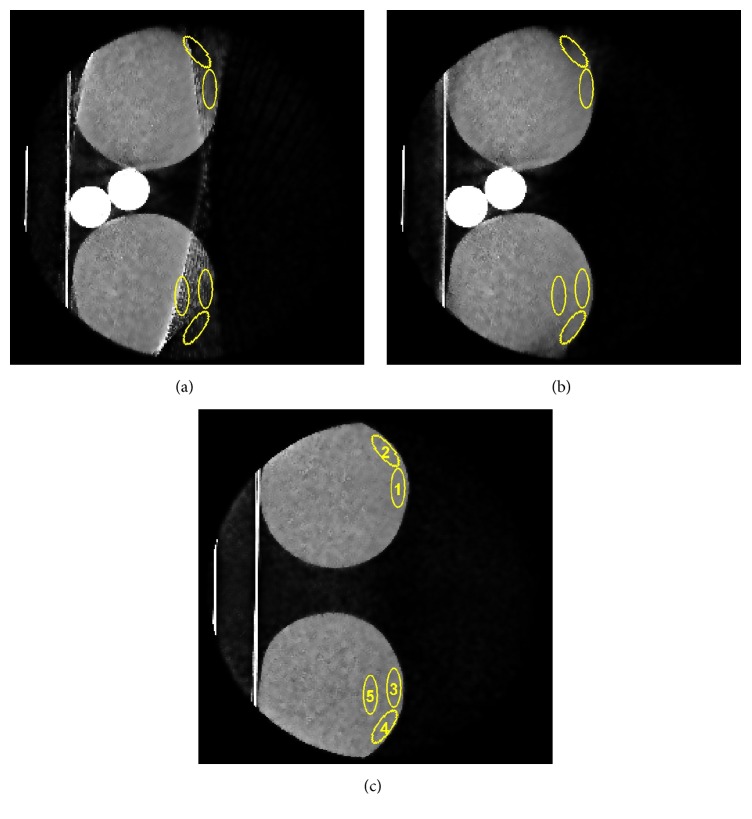
(a), (b), and (c) show 5 ROIs in the exterior regions of the bottles which are used for comparison reconstructed projections. The ROIs are evaluated for one slice in the uncorrected (a), corrected (b), and ground truth (c) data. Window level: [−1000, 1000].

For quantitative comparison, five regions of interest (ROIs) are placed in the exterior regions of the bottles (Figure 13). The measurements are shown in Table 1. The table compares the measurements of the HU values within the ROIs for the reconstructions of the uncorrected projections, the corrected projections, and the ground truth.

For the corrected reconstruction, we can observe that the mean values of ROIs move closer to the corresponding ground truth values. This change occurs because the previously truncated parts of the phantom are now partially restored at the positions of the ROIs. Now, the material of the phantom is more consistently measured inside the ROIs instead of air in the truncated case.

Furthermore, the values of the standard deviation are reduced. This shows that the values within the ROI in the corrected data are more homogeneous and outliers, which would increase the standard deviation, have been eliminated. The saturation artifacts cause very high maximum values on the truncated edge of the lower bottle. These artifacts are corrected with the Kinect-based correction tool.

The corrected data shows significantly improved reconstruction results. The visualization of the absolute differences between both uncorrected and corrected data and the ground truth (see Figures 11(e) and 11(f)) backs up our measurements. We observe that the differences are lower for almost all regions. Furthermore, the figures show that, apart from artifact correction inside the knee phantoms, artifacts caused by truncation between the two phantoms were also reduced.

## 5. Discussion

The results have shown that the Kinect-based correction of saturation in cone-beam CT is a feasible approach for reducing artifacts in saturated scans. Lost surface information, especially at the front side of the knee phantoms, was restored. Furthermore, noise and overexposure artifacts were reduced through the correction of the projections.

Overexposure not only exclusively occurs in C-arm CT imaging but also occurs in other systems such as multidetector CT (MDCT). One factor that makes overexposure compensation easier in MDCT is the higher dynamic range of 20 bits [20], which generally leads to less severe artifacts. Furthermore, bowtie filters and tube current modulation can be utilized to reduce radiation dosage in the exterior regions of the scanned object [21–23]. In C-arm CT, overexposure artifacts are mostly tackled after image acquisition, as bowtie filters are linked to reduced detector efficiency [24] and overexposure of the detector is often even intentionally caused to tackle image quality limitations due to the limited dynamic range [9, 25, 26].

Fully leveraging the 200° raw data acquisition of the C-arm CT around the knees might allow for better correction results in algorithmic approaches than the baseline considered in this paper. ROI reconstruction [27–29] or iterative reconstruction [30] could be utilized for this approach. Severe truncation, however, is still unlikely to be fully corrected [30]. In this context, it should be noted that the proposed method can, in principle, be used in combination with any other correction method. Using the additional surface information could be used for regularization which would likely lead to further performance improvements.

Additional considerations would have to be made, if the overexposure occurred in the bone, for example, the patella. In this case, the normalization factor would be based on the bone density. Instead of the skin tissue, the bone would be expanded until the outer surface, which would cause correction errors. The first likely occurrence of overexposure is to be expected in the skin tissue right next to the bones. The extrapolation of the skin tissue based on the values of the neighboring bone tissue can algorithmically be avoided. If the values of the last nonoverexposed pixels are significantly higher than expected for skin tissue, the normalization factor can be adjusted according to nearby or typical skin tissue values.

In the B-spline interpolation we observe inaccuracies of the edge detection of the Kinect camera. For a bigger field of view problems may arise in the correction of the outer edges. However, saturation artifacts are usually only expected at front side and back side of the knees for patient scans. For these regions we can acquire reliable information with the Kinect camera.

Choi et al. [31] proposed an approach for motion correction in weight-bearing knee scans. However, it is still necessary to correct for overexposure artifacts. A depth camera-based solution offers the possibility of a temporally synchronized correction of overexposure artifacts, because the depth information is captured in real-time and continuously throughout the complete scanning procedure.

## 6. Outlook

The experiments in our research aim to demonstrate the general feasibility of the correction method. For this, we focus on supine scans of the human knees. However, the design of the method is not restricted to supine scans and could in principle also be used for weight-bearing scans of the knees in real-world scenarios.

For this, we propose using two Kinect sensors to gather surface information for all relevant angles. The design of the cross-calibration phantom allows the simultaneous cross-calibration of two Kinect sensors with the C-arm CT. By capturing the surface areas close to the patella and popliteus with two separate cameras, closed B-spline curves can directly be calculated from the merged surface data and used for saturation correction. By using this approach, no further estimations for the back side of the object have to be made and more accurate results are to be expected.

In this paper, we analyzed the new correction approach in isolation. The correction method could be combined with other recent algorithmic approaches to leverage their respective benefits. In future experiments, the performance improvements of the artifact correction for combined approaches could therefore be investigated in detail.

In order to use proposed approach in real-world scenarios, the accuracy of the cross-calibration is of high importance and can be improved through more precise manufacturing. Design improvements could be achieved by evaluating the cross-calibration accuracy for different positions, sizes, and numbers of spheres. Transparent materials are usually not detectable by the depth camera and could be used for the sphere-carrying rods to improve the segmentation accuracy.

Besides qualitative improvements in the phantom design, the procedure could be improved algorithmically. In the experiments, only depth features from the Kinect sensor are used for the calibration. By making use of the additional RGB data gathered by the Kinect, the accuracy of the cross-calibration could be further enhanced.

Big improvements in processing time can be made in the projection correction. The main source of computing time derives from the B-spline curve interpolation and calculation of line integrals along the X-rays through the object. This type of calculation is one of the basic routines on a GPU and could be performed by providing the graphics card with the 3D points and projection geometry [32].

The sphere segmentation was performed semiautomatically by first clicking on the individual spheres in a predefined order. In future, the spheres could be detected in the RGB image automatically, based on their color.

## 7. Summary

When scanning knees, the limited dynamic range of the detector causes saturation artifacts in the reconstructed volumes. As these artifacts affect the surface regions of the scanned object, the idea for the correction method is to additionally use a Kinect camera to locate the surface of the object in 3D.

In order to use these surface points for the correction of CT images, we develop a procedure for cross-calibration between the camera and the C-arm CT. For cross-calibration we use a PDS-2 calibration phantom and attached a structure that is detectable with the Kinect camera.

After the cross-calibration, a projection-based saturation correction is performed where all detector lines are successively corrected within the projections. With the C-arm geometry, we determine the 3D points where the X-rays entered and exited the knee and calculate the length of the X-ray through the knee with these points. Ultimately, we use these calculated lengths for smooth extrapolation of the boundary of the object in the overexposed regions.

The reconstruction results show that the projection-based correction itself yields clear improvements to the noncorrected data. The boundaries of both knee phantoms are extrapolated to their correct position and overexposure artifacts are significantly reduced.

Potentially arising problems due to limited edge detection and the different tissue densities in the knees are also considered.

Possible future work includes the usage of a second Kinect camera for weight-bearing scans and a GPU-based calculation of the intersection lengths. The sphere segmentation could be automated by identifying the spheres based on their color. Furthermore, a temporally synchronized correction approach could be applied in current research projects.

## Figures and Tables

**Table 1:** Comparison of the measurements of the ROIS shown in Figure 13.

Type	ROI	Mean	Std dev	Min	Max
Uncorrected	1	−478	111	−727	−92
	2	−715	319	−988	364
	3	−568	191	−861	68
	4	−806	89	−922	−455
	5	−128	458	−793	1333

Corrected	1	−245	81	−598	−120
	2	−528	143	−873	−219
	3	−133	63	−296	28
	4	−284	72	−559	−147
	5	−139	59	−290	−15

Ground truth	1	−60	34	−181	36
	2	−30	51	−157	80
	3	−99	46	−202	27
	4	−43	51	−195	122
	5	−52	61	−181	173

## Appendix

### A. Mathematical Formulas

#### A.1. Line-Line Intersection

All direction and normal vectors of lines in the following equations shall be considered as unit vectors. Two-dimensional lines can be represented by a point **p**_line_ on the line and a normal vector **n**_line_ perpendicular to that line. The distance between a point **g** and a line defined by **p**_line_ and **n**_line_ is

(A.1) dplg,pline,nline=g−plineTnline=g−plineTnlinenlineTg−pline.

The sum of squared distances to more than one line is

(A.2) wg∑idplg,pline,i,nline,i2=∑ig−pline,iTnline,inline,iTg−pline,i.

To find the closest mutual point, that is, the minimum of function *w*(**g**), the equation has to be differentiated with respect to **g** and the result has to be set equal to the zero vector. This leads to

(A.3) $$\begin{array}{c} g={\left(\;\sum_{i}n_{line,i}n_{line,i}^{T}\;\right)}^{-1}\cdot \left(\;\underset{i}{\sum }n_{line,i}n_{line,i}^{T}p_{line,i}\;\right). \end{array}$$

The equation

(A.4) $$\begin{array}{c} I=n_{line}n_{line}^{T}+v_{line}v_{line}^{T} \end{array}$$

with the 2 × 2 identity matrix **I** and normalized direction vector **v**_line_ is introduced and proved for the two-dimensional case. Multiplying both sides of (A.4) with any direction or normal vector **v**_line_ or **n**_line_ leads to

(A.5) vlinenlinenlineTvline+vlinevlineTvline=nline·0+vline·1=vline,nlinenlinenlineTnline+vlinevlineTnline=nline·1+vline·0=nline,

which is always true. Equation (A.4) is rearranged to

(A.6) $$\begin{array}{c} n_{line}n_{line}^{T}=I-v_{line}v_{line}^{T} \end{array}$$

and used to modify (A.3), which leads to the final solution for **g**:

(A.7) g=∑iI−vline,ivline,iT−1·∑iI−vline,ivline,iTpline,i.

The calculation of **g** in three dimensions is very similar. Equations (A.3) and (A.4) are sufficient in 2D cases, because the two orthogonal vectors **v**_line_ and **n**_line_ span the 2D space. The calculation of the mutually closest point in 3D has to take into account a second normal vector **m**_line_ but is analog to the 2D case apart from that. The vectors **v**_line_, **m**_line_, and **n**_line_ are pairwise orthogonal and therefore span the 3D space. For three-dimensional lines, (A.3) and (A.4) contain the sum (**n**_line_ **n**_line_^T^ + **m**_line_ **m**_line_^T^) instead of (**n**_line_ **n**_line_^T^) and **I** now is the 3 × 3 unity matrix.

The mutually closest point is still calculated as described in (A.7), because the sum **n**_line_ **n**_line_^T^ + **m**_line_ **m**_line_^T^ is replaced in the same way as in the 2D case:

(A.8) $$\begin{array}{c} n_{line}n_{line}^{T}+m_{line}m_{line}^{T}=I-v_{line}v_{line}^{T}. \end{array}$$

#### A.2. Coordinate System Transformation

The first step of the transformation between both coordinate systems is a coordinate system rotation. The rotation matrix is calculated by using the matrix rotation formula

(A.9) $$\begin{array}{c} WC=D, \end{array}$$

where **W** is the 3 × 3 rotation matrix that is used to rotate the orthogonal axis vectors of the Kinect coordinate system onto the corresponding axis vectors of the Zeego C-arm coordinate system. **C** is the matrix containing the unit direction vectors of the sphere mount axes in the Kinect coordinate system. **D** is the matrix containing the unit direction vectors of the sphere mount axes in the Zeego coordinate system. Using (A.9), the direction vectors of **C** shall be rotated onto the direction vectors of **D**. The rotation matrix **W** can be obtained by calculating the matrix inverse **C** ^−1^ and rearranging the equation to

(A.10) $$\begin{array}{c} W=DC^{-1}. \end{array}$$

After rotating the points with the rotation matrix **W**, the final step of the coordinate system transformation is the translation of the coordinate system origin to the center of the PDS-2 phantom which amounts to 110 mm on the *z*-axis:

(A.11) $$\begin{array}{c} t^{Origins}=\left[\begin{array}{c} 0\\ 0\\ -110 \end{array}\right]. \end{array}$$

Subsequently, the complete transformation is

(A.12) $$\begin{array}{c} p^{Zeego}=Wp^{{Kinect}^{\prime }}+t^{Origins}. \end{array}$$
